# Editorial

**Published:** 1861-09

**Authors:** 


### Editorial.

### AMERICAN DENTAL CONVENTION.

The Register was not represented at the late meeting of the Convention at New Haven. For this we are sorry ; but, as we have fallen on peculiar times, we hope our readers will not regard the fact of our absence as an evidence of a lack of interest. We have no full or extended report of the proceedings ; but if any of our brethren have, we will, with their consent, of course, borrow, and lay before our readers, the whole report, or at least as much of it as may prove of special interest.

We are glad to have evidence that our friends have not all forgotten us. Prof. Buckingham, and somebody else, sent us some numbers of the "Journal and Courier," and of the "Herald and Journal," containing reports of the proceedings of the Convention. From these we learn that but sixty-eight dentists were present—a small number, but as many as could be expected under the circumstances. Dr. John Allen was elected President, a position fairly and honorably earned by his earnestness in and devotion to the advancement and improvement of mechanical dentistry. Dr. J. D. White was chosen Vice-President, a position he merited by breaking over his former habits (of staying away) and coming up to the help of the Convention in the hour of its weakness and trial. We felt relieved when we found friend "J. D." among the "members present,"—felt better on finding him Vice-President,"—better still, that he pitched right into the discussions with his usual ability, and with all the zeal incident to new conversion. We hope he will, having taken the first step, carry out the colored Calvinist's idea of the "perseverance of the saints"—"take hold—*hold on*—and never let go." Dr. F. Searle was elected Recording Secretary ; Dr. B. T. Whitney, Corresponding Secretary ; and Dr. J. T. Metcalf, Treasurer,—good men and true, all—no round men put into square holes.

A letter, in reference to a proposed "World's Dental Convention," was received from the "College of Dentists," England ; and an answer was returned by a committee, specially appointed for the purpose, consisting of Drs. T. L. Buckingham, J. J. Wetherbee, and A. McIlroy.

The general divisions of the "Order of Business," as adopted by the Convention, are "Etiquette, Surgical Dentistry, Mechanical Dentistry, and Unfinished and Miscellaneous Business." Various subdivisions were proposed under each of the first three, which it is not our present purpose to notice. "When shall we insert pivot teeth?" placed under the mechanical department, appears to us more *surgical* than otherwise, even though the invention of the teeth may be mechanical.

Dr. Allen read a paper on the "Causes which retard the progress of Dentistry," and Prof. W. H. Atkinson read two,—the first entitled, "What lack I yet?" and the second, "Does being imply the right to live?" Without seeing the papers, we venture to state, in reference to the first question, that our friend A. lacks much less than many others.

"Your wants, dear sir, will seem but small,
When they're compared with mine;
My single want outweighs them all—
I want a soul like thine."

The second question is beyond our capacity, hence, we will not risk an answer, but refer the reader to the paper. Dr. Burras also read a paper on "Mastication and the Articulation of Artificial Dentures." All of these papers we hope to see.

A committee was appointed with reference to the appointment of dentists in the army and navy of the United States. An order of exercises for the next meeting was adopted, which we will lay before our readers in due time. A committee, specially appointed, reported a constitution, which we infer was adopted. Trenton Falls, New York, was selected as the place for the next meeting. Votes of thanks were abundantly given for specific favors as abundantly received. And, after it got ready, on the fourth day of its session, the Convention adjourned to meet again, the second Tuesday of August, 1862.

We infer that the meeting was harmonious, and every way pleasant. We hope to give the proceedings in full, hereafter; and we may take occasion to notice some of the positions assumed.

W.

### DENTAL HOSPITALS.

We learn by the London Dental Review, that our professional brethren of London and its vicinity are making a move for the establishment of a National Dental Hospital.

The object and scope of the hospital is declared to be not merely metropolitan, but national ; one for the benefit of the whole kingdom, to be for the relief of those poor persons who can not afford to pay a dentist's fee, when suffering from dental diseases. It is expected to receive patients and subscriptions from all parts of the country.

The following resolution was offered, discussed and adopted, by a meeting of the dentists having the matter in charge, viz : "That an institution to be called the National Dental Hospital be now established. The hospital to provide gratuitous and efficient aid to the poor in diseases of the teeth, and to include means for instruction to students, preparing for dental practice, as members of the College of Dentists."

This movement is based upon a conviction of the fact, that the profession has made such attainments as will make it eminently valuable to all classes of society ; indeed so much so that none should be without its aid. Society, as a whole, has a right to demand such aid. Such institutions, in addition to being instrumental in the alleviation of human suffering, will be of incalculable advantage for the further development of professional attainments, and especially to those who are seeking knowledge.

In the establishment of dental hospitals our English brethren are in advance of us. It is true, we first established Dental Colleges ; but they, too, in London have established an institution of this character ; and one, we should think, well calculated to accomplish the object of its institution.

They did not stop with this, but immediately set about taking the next very natural step, viz : the establishment of dental hospitals. We have made but a feeble effort in this direction, in the establishment of dental infirmaries in connection with our colleges. Only one idea, however, is prominent in these, viz : that of giving instruction to the students, leaving out of view almost entirely the highest good of the patients. They were first instituted avowedly for furnishing means of instruction to students ; they are kept open only during the college session.

We think it very desirable that something more than this should be done here, as well as in London. It has been suggested that a dental department in the regular hospitals might accomplish the desired objects. While it is true that something might be accomplished in this way, it is very evident that the greatest good could not thus be accomplished. In the ordinary hospitals, there are very few patients who are in condition for dental treatment. It might be desirable to have a dentist connected with every general hospital; but then we do not conceive that the aims and objects of a dental hospital could be efficiently carried out in such institutions.

Dental hospitals should be established upon such a basis and plan as would most fully meet the wants of those who are unable to pay the ordinary fees for dental operations; and at the same time afford facilities for improvement to those seeking attainments in the practical and scientific departments of the profession.

We may refer to this subject again. T.

